# A Cutaneous Clue to Prolonged Fever: Erythema Induratum of Bazin in a Tuberculosis-Endemic Region

**DOI:** 10.7759/cureus.114905

**Published:** 2026-08-21

**Authors:** Biswajit Mohanty, Niranjan Mohapatra, Jayant Panda, Gudly Nanda

**Affiliations:** 1 General Medicine, Sriram Chandra Bhanja Medical College Hospital, Cuttack, IND; 2 Neuroimaging and Interventional Radiology, National Institute of Mental Health and Neurosciences, Bangalore, IND

**Keywords:** bazin disease, cutaneous tuberculosis, erythema induratum, nodular rash, panniculitis, tuberculid

## Abstract

Erythema induratum of Bazin (EIB) is a rare form of cutaneous tuberculosis characterised by recurrent nodular lesions, predominantly affecting the lower extremities. It is considered a hypersensitivity reaction to Mycobacterium tuberculosis rather than a direct infection, hence the term "tuberculid". Due to its nonspecific clinical presentation, it often mimics other dermatological and systemic conditions, leading to diagnostic challenges. We report a case of a 40-year-old woman presenting with prolonged low-grade fever, weight loss, and multiple tender nodular lesions over the lower limbs and forearms. Laboratory evaluation revealed anaemia of chronic disease and elevated inflammatory markers, while autoimmune and infectious workup was negative. Histopathological examination demonstrated lobular panniculitis with lymphohistiocytic infiltrates and granulomatous inflammation along with vasculitis, consistent with EIB. The patient was initiated on anti-tubercular therapy, resulting in complete resolution of symptoms and lesions. This case highlights the importance of considering cutaneous tuberculosis in patients with persistent nodular lesions, particularly in endemic regions, and underscores the diagnostic value of histopathology.

## Introduction

Erythema induratum of Bazin (EIB) is an uncommon manifestation of cutaneous tuberculosis, first described by Ernest Bazin in 1861. It is classified as a tuberculid, a group of dermatoses arising from delayed hypersensitivity reactions to Mycobacterium tuberculosis antigens in individuals with intact cell-mediated immunity, rather than from direct mycobacterial invasion of the skin [1,2]. Clinically, it manifests as recurrent, tender, indurated nodules on the lower extremities, predominantly affecting middle-aged women, and may ulcerate and heal with scarring [3].

The diagnosis is often challenging due to the nonspecific presentation and histological overlap with conditions such as erythema nodosum, nodular vasculitis (NV), and other forms of panniculitis [4]. Histopathology showing lobular panniculitis with granulomatous inflammation and foci of vasculitis remains the diagnostic cornerstone [5]. Microbiological confirmation is frequently elusive owing to the paucibacillary nature of the disease, necessitating clinicopathological correlation [6].

We report a case of EIB presenting with prolonged fever and nodular rash, emphasising the importance of clinical suspicion, histopathological evaluation, and interferon-gamma release assay (IGRA) in establishing the diagnosis in a tuberculosis-endemic setting.

## Case presentation

A 40-year-old woman from rural Odisha, a farmer by occupation, presented with low-grade fever for four months, with an evening rise in temperature over the past month. She also reported multiple nodular skin lesions over both lower limbs (Figures 1A, 1B) for three months, initially painful, with subsequent development of similar lesions over both forearms (Figure 1C). The patient also noted significant unintentional weight loss. There was no history of cough, joint pain, oral ulcers, diarrhoea, tuberculosis exposure, or prior medication use. She had no known comorbidities. On examination, multiple erythematous nodular lesions of varying sizes were observed over the ventral and dorsal aspects of both legs, along with two similar lesions over the forearms. Some lesions were tender, while others were indurated. There was no evidence of ulceration/discharge. Systemic examination was otherwise unremarkable. 

**Figure 1:**
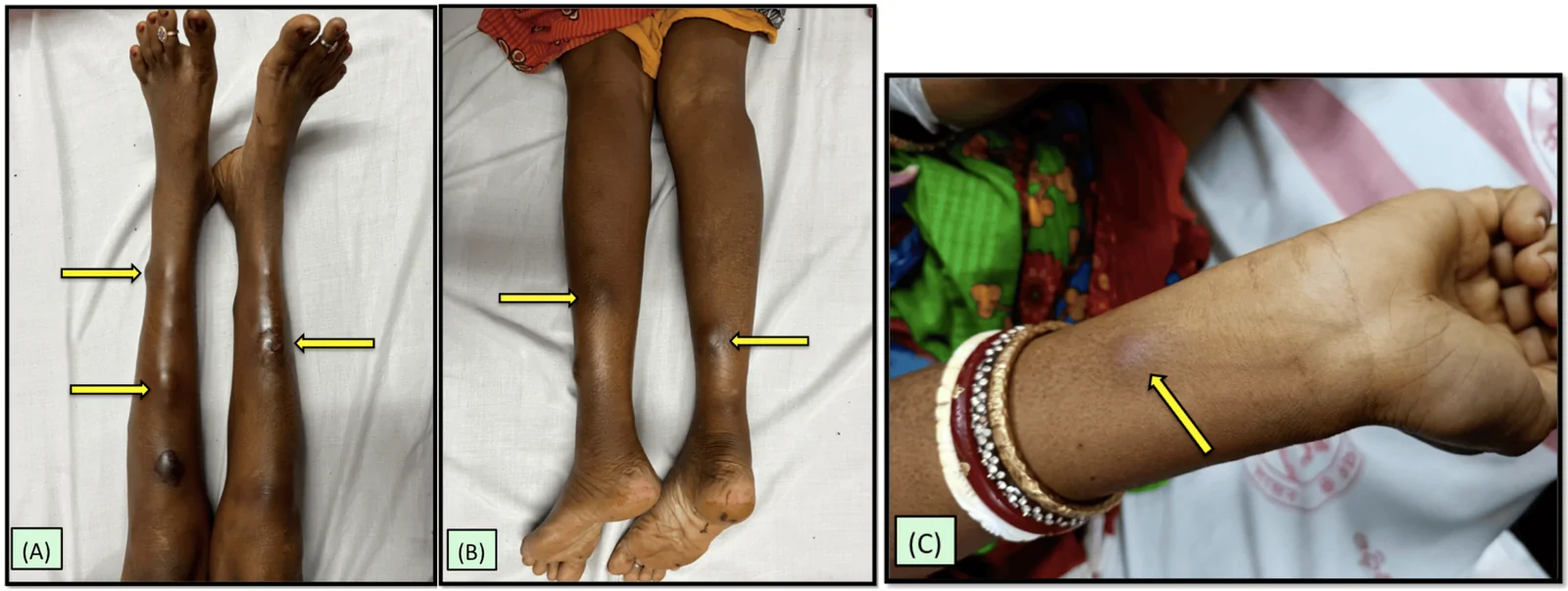
Erythematous nodular lesions over bilateral lower limbs: ventral aspect (A), dorsal aspect (B), and right forearm (C)

Laboratory investigations (Table 1) revealed microcytic hypochromic anaemia and a persistently elevated erythrocyte sedimentation rate. Liver and renal function tests were within normal limits. Chest imaging including chest X-ray and high-resolution computed tomography was normal. Abdominal imaging revealed no significant abnormality. Antinuclear antibodies were negative, and serum angiotensin-converting enzyme levels were within normal limits. Direct Coombs test was negative. Iron studies were suggestive of anaemia of chronic disease. Dermatological consultation was sought, following which a skin biopsy from a representative lesion revealed lobular panniculitis with dense lymphohistiocytic infiltrates involving the subcutaneous fat. The dermis showed perivascular chronic inflammatory infiltrates composed of lymphocytes and plasma cells, along with clusters of histiocytes forming vague granulomas and foci of vasculitis (Figure 2). AFB staining for acid-fast bacilli was negative. These findings were consistent with EIB. Subsequently, an IGRA using the QuantiFERON-TB Gold was performed, which yielded a positive result.

**Table 1:** Relevant laboratory investigations TLC: Total Leucocyte Count; ESR: Erythrocyte Sedimentation Rate; CRP: C-Reactive Protein; TIBC: Total Iron Binding Capacity; ACE: Angiotensin-Converting Enzyme

Investigation	Result	Normal Value
Haemoglobin (g/dL)	8.4	11.0 - 17.0
TLC (x10^3^/Cumm)	8.2	4.0 - 11.0
Platelets (x10^3^/Cumm)	240	150 - 450
Neutrophils (%)	68.3	40 - 75
Lymphocytes (%)	26.5	20 - 45
Monocytes (%)	3.4	2.0 - 10.0
Eosinophils (%)	1.6	0.0 - 6.0
Basophils (%)	0.2	0.0 - 1.0
ESR (mm/hour)	80	< 20
Serum Urea (mg/dL)	36	15 - 40
Serum Creatinine (mg/dL)	1.1	0.5 - 1.2
CRP (mg/L)	8.2	< 6
Serum Iron (mcg/dL)	35	50 – 150
Serum Ferritin (ng/mL)	300	15 - 200
TIBC (mcg/dL)	225	250 - 450
Transferrin Saturation (%)	15	20 - 45
Serum ACE (U/L)	45	23 - 57
Serum Sodium (mEq/L)	138	135 – 145
Serum Potassium (mEq/L)	3.8	3.5 – 4.5

**Figure 2:**
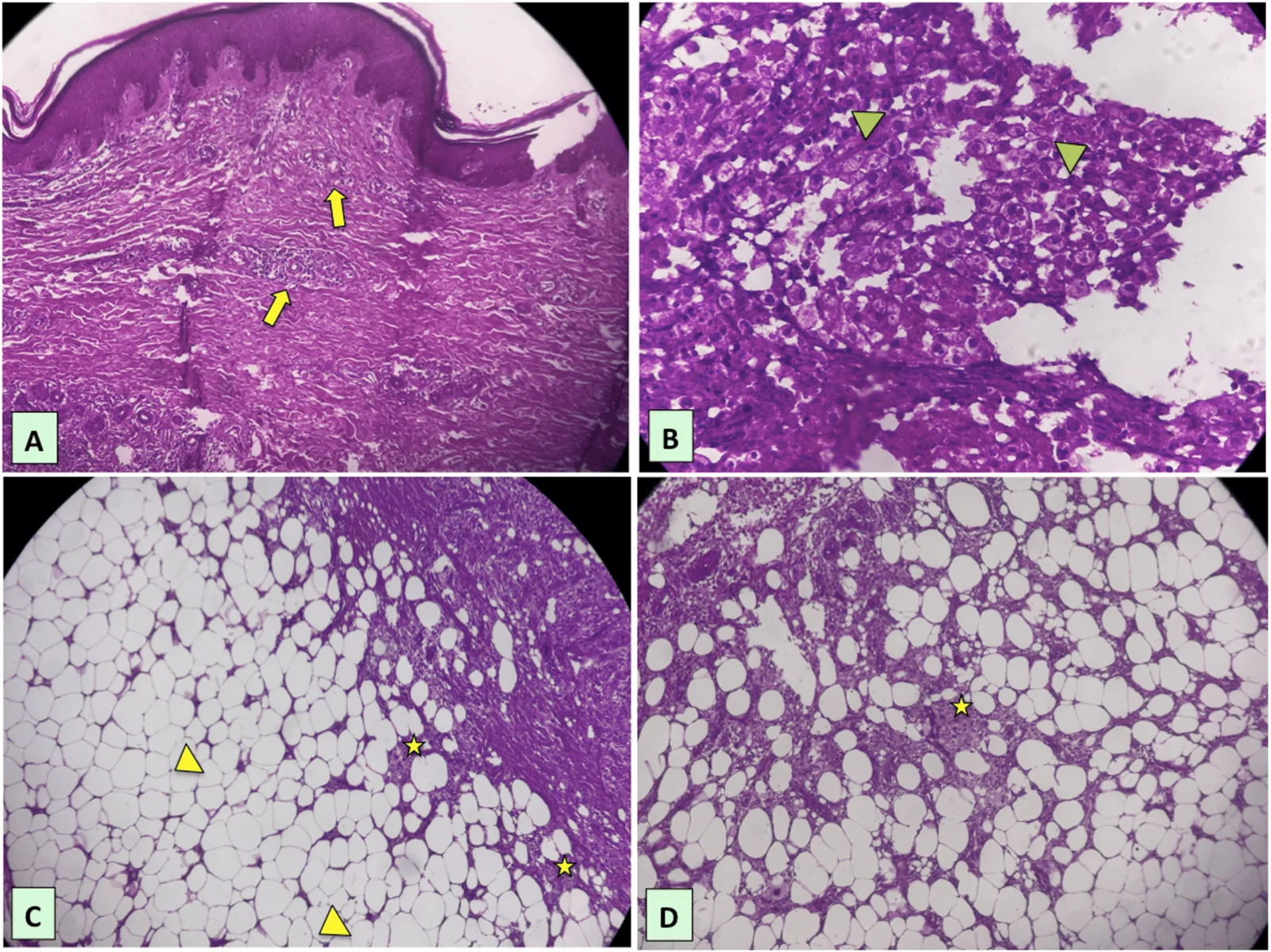
Histopathology of skin biopsy: (A) Perivascular chronic inflammatory infiltrates composed of lymphocytes in the dermis (yellow arrows), (B) Cluster of histiocytes forming vague granulomas (green arrowheads), and (C) and (D) Lobular panniculitis with dense lymphohistiocytic infiltrates (yellow asterisk) in the subcutaneous fat vs. normal fat globules and septa (yellow arrowheads) (H&E, 400X)

In view of the diagnosis of EIB, anti-tubercular therapy (ATT) was commenced as per national guidelines. Within one month of therapy, the fever subsided, and the skin lesions showed significant improvement. Complete resolution of lesions was noted over subsequent follow-up visits. The patient completed the full course of therapy without recurrence.

## Discussion

EIB is a rare form of cutaneous tuberculosis predominantly affecting middle-aged women and pathogenetically driven by a Type IV (delayed-type) hypersensitivity reaction to antigens of Mycobacterium tuberculosis. Unlike forms of cutaneous tuberculosis involving direct bacterial seeding of the skin, EIB represents an immunologically mediated reaction to haematogenously disseminated bacillary antigens in a host with strong cellular immunity [1,2]. The disease belongs to the tuberculid spectrum, which also includes papulonecrotic tuberculid and lichen scrofulosorum [3]. Contemporary literature has expanded the recognised demographic beyond young women with circulatory insufficiency to include immunocompetent adults in high-burden regions such as the Indian subcontinent [4].

The classical clinical presentation consists of recurrent, tender, indurated, erythematous-to-violaceous nodules on the calves and lower legs, which may ulcerate and heal with atrophic, pigmented scarring [5]. The involvement of the forearms, as noted in our patient, represents an atypical and uncommon anatomical distribution. Disseminated or atypical EIB has been described in cases of heightened mycobacterial antigen load and in patients residing in tuberculosis-endemic regions, suggesting that both systemic antigen burden and local microvascular factors govern the distribution of lesions [6]. The co-existence of prolonged low-grade fever, constitutional weight loss, and anaemia of chronic disease in our patient expanded the differential diagnosis to include erythema nodosum, lymphoma, sarcoidosis, systemic vasculitis, connective tissue diseases, and other recognised causes of prolonged fever, significantly delaying the recognition of EIB as the unifying diagnosis [7].

The pathological hallmark of EIB is lobular panniculitis, inflammation predominantly involving the fat lobules of the subcutaneous tissue, accompanied by lymphohistiocytic and plasmacytic infiltration, vasculitis of small- and medium-sized adipose tissue vessels, and granuloma formation [4,5]. Caseous necrosis, while classic, is not invariably present, and its absence should not preclude the diagnosis. The histopathological distinction between EIB and NV, a morphologically similar entity unrelated to tuberculosis, has been debated in the literature. Both share lobular panniculitis with vasculitis; however, EIB is preferentially diagnosed when indirect evidence of mycobacterial sensitisation is present, such as IGRA positivity, a strongly reactive tuberculin skin test (TST), or a favourable response to anti-tubercular therapy [4,8].

Microbiological confirmation of EIB via acid-fast bacilli smear or culture from biopsy specimens is achieved in fewer than 10% of cases, owing to the paucibacillary nature of the disease [6]. Polymerase chain reaction-based detection of mycobacterial DNA in biopsy tissue offers improved sensitivity and represents a valuable adjunctive tool in diagnostically challenging or culture-negative cases [6]. In the present case, the QuantiFERON-TB Gold IGRA was reactive, providing strong indirect immunological evidence of mycobacterial sensitisation consistent with the tuberculid mechanism. Multiple studies have validated IGRA as a highly useful ancillary test in EIB, with one Korean series reporting 100% positivity in 22 histologically confirmed cases [8]. IGRA is preferred over the TST in BCG-vaccinated populations, as it avoids false positivity attributable to prior vaccination and environmental mycobacterial exposure [9]. However, IGRA positivity reflects latent or prior tuberculosis sensitisation and must always be integrated with clinical and histological data rather than interpreted in isolation.

India carries the highest absolute burden of tuberculosis globally, accounting for approximately 26% of incident TB cases worldwide, according to the WHO Global Tuberculosis Report 2024 [10]. In such endemic settings, extrapulmonary and cutaneous forms of tuberculosis, including tuberculids like EIB, merit early consideration even when conventional microbiological proof is lacking. A normal chest and abdominal imaging, as observed in our patient, does not exclude an underlying or prior mycobacterial infection, since tuberculids arise in the context of latent rather than active pulmonary disease [3,11]. Clinicians in endemic regions must therefore maintain a high index of suspicion for EIB when evaluating patients with recurrent nodular lower-limb lesions, particularly when accompanied by constitutional symptoms and elevated inflammatory markers.

The treatment of EIB follows the standard ATT regimen for extrapulmonary tuberculosis as per national guidelines [11]. Favourable clinical response to ATT - fever resolution within one month and complete clearance of skin lesions over the full treatment course, as observed in our patient - reinforces the diagnosis and may serve as a therapeutic test in histologically equivocal cases [7,11]. Non-steroidal anti-inflammatory drugs may be used as adjuncts for symptom relief in the early treatment phase. Recurrence following completion of ATT is uncommon in immunocompetent individuals; however, prolonged follow-up is recommended to detect any relapse promptly.

This case underscores several important clinical lessons. First, EIB should be included in the differential diagnosis of prolonged fever whenever persistent nodular skin lesions are present, particularly in tuberculosis-endemic regions. Second, the absence of pulmonary tuberculosis on chest imaging does not exclude the diagnosis of EIB. Third, a prompt dermatological opinion with histopathological examination of skin biopsy demonstrating lobular panniculitis with granulomata and vasculitis is indispensable for diagnosis confirmation. Fourth, IGRA is a valuable ancillary test and should be performed when EIB is clinically or histologically suspected [8]. Fifth, prompt initiation of ATT prevents the unnecessary diagnostic odyssey often associated with prolonged fever and leads to complete and durable clinical resolution.

## Conclusions

EIB is an uncommon but clinically significant manifestation of cutaneous tuberculosis that can present with prolonged fever, particularly in endemic regions. Its nonspecific clinical presentation frequently leads to delayed or missed diagnosis. A high index of suspicion, combined with skin biopsy demonstrating lobular panniculitis, granulomatous inflammation and vasculitis, is essential for timely diagnosis. IGRA provides important immunological corroboration. Early institution of standard anti-tubercular therapy leads to complete resolution of both systemic and cutaneous manifestations. Clinicians practising in tuberculosis-endemic areas must remain vigilant to this uncommon but eminently treatable condition.
